# Relevance of vitamin D deficiency in patients with chronic autoimmune atrophic gastritis: a prospective study

**DOI:** 10.1186/s12876-018-0901-0

**Published:** 2018-11-08

**Authors:** Sara Massironi, Federica Cavalcoli, Alessandra Zilli, Alessandro Del Gobbo, Clorinda Ciafardini, Susanna Bernasconi, Irene Felicetta, Dario Conte, Maddalena Peracchi

**Affiliations:** 1 Gastroenterology and Endoscopy Unit, Fondazione IRCCS Ca’ Granda Ospedale Maggiore Policlinico, Milan, Italy; 2 Department of Pathophysiology and Transplantation, Università degli Studi di Milano, Milan, Italy; 3 Division of Pathology, Fondazione IRCCS Ca’ Granda Ospedale Maggiore Policlinico, 20122 Milan, Italy; 4 Laboratory of Clinical Chemistry and Microbiology, Fondazione IRCCS Ca’ Granda Ospedale Maggiore Policlinico, Milan, Italy

**Keywords:** Chronic autoimmune atrophic gastritis, Vitamin D deficiency, Gastric carcinoid, Bone health, Osteoporosis

## Abstract

**Background:**

Chronic autoimmune atrophic gastritis (CAAG) is an autoimmune disease characterized by hypo/achlorhydria. A role of CAAG in the pathogenesis of nutritional deficiencies has been reported, therefore we hypothesized a possible association between CAAG and 25-OH-Vitamin D [25(OH)D] deficiency. Aim of the present study is to evaluate the prevalence of 25(OH)D deficiency in CAAG patients. Methods: 87 CAAG patients (71 females; mean age 63.5 ± 12.8 years) followed at our Centre from January 2012 to July 2015 were consecutively evaluated. 25(OH)D, vitamin B_12_, parathormone, and calcium were measured in all the CAAG patients. The results were compared with a control group of 1232 healthy subjects.

**Results:**

In the CAAG group the mean 25(OH)D levels were significantly lower than in the control group (18.8 vs. 27.0 ng/ml, *p* < 0.0001). 25(OH)D levels < 20 ng/ml was observed in 57 patients, while levels < 12.5 ng/ml in 27 patients. A significant correlation between vitamin B_12_ values at diagnosis and 25(OH)D levels was observed (r_s_ = 0.25, *p* = 0.01). Interestingly, the CAAG patients with moderate/severe gastric atrophy had lower 25(OH)D values as compared to those with mild atrophy (11.8 vs. 20 ng/ml; *p* = 0.0047). Moreover, the 25(OH)D levels were significantly lower in CAAG patients with gastric carcinoid as compared to those without gastric carcinoid (11.8 vs. 19.8 ng/ml; *p* = 0,0041).

**Conclusion:**

Data from the present study showed a significant reduction of 25(OH)D levels in CAAG patients and a possible impairment of vitamin D absorption in CAAG may be postulated. Any implication to the genesis of gastric carcinoids remains to be elucidated.

## Background

Chronic autoimmune atrophic gastritis (CAAG) is an immune-mediated inflammatory condition involving the parietal cells in the gastric fundus and body [1, 2]. Parietal cells secrete intrinsic factor and hydrochloric acid, via the H+/K+ adenosine triphosphatase proton pump, and are the main determinant of gastric acidification. The chronic inflammation results in mucosal atrophy with a progressive destruction and ultimate complete loss of parietal cells [3], increase in gastric pH, hypergastrinemia and intrinsic factor deficiency [4].

Intrinsic factor is a co-factor essential for vitamin B12 absorption in the terminal ileum and its deficiency in CAAG leads to vitamin B12 deficiency [5, 6]. In this setting, also iron insufficiency and iron deficiency anemia have been recently observed, especially in premenopausal women [7, 8]. The relevance of physiological gastric acid secretion for iron absorption has been suggested by several authors. In fact, nutritional iron is usually bound to proteins and requires the gastric acidification for its solubilization and uptake [9]. Furthermore, an increased incidence of different vitamins and micronutrients deficiency (e.g. ascorbic acid C and folate) in CAAG was reported [10–12]. It has been postulated that hypochlorhydria and bacterial overgrowth in CAAG patients may cause a decreased absorption or increased loss of nutrients in the stomach [11].

A higher incidence of osteopenia and osteoporosis in conditions determining gastric hypochlorhydria, such as gastric resection [13], proton pump inhibitors (PPIs) therapy and CAAG [14–17] has been suggested. However, the pathogenic mechanism leading to these alterations has not been clarified yet. A few studies have reported about a reduced absorption of calcium in patients affected by CAAG [18, 19], while a study by Eastell et al. did not find any significant differences in calcium absorption in CAAG patients as compared with controls [20]. Interestingly*,* two studies have recently reported an association between CAAG and vitamin D deficiency [21, 22] and a possible role of vitamin D deficiency in the increased risk of osteopenia/osteoporosis in these patients has been proposed.

Therefore, a possible role of CAAG in determining a vitamin D deficiency could be hypothesized, however, to date there is lack of studies investigating the relevance of vitamin D deficiency in CAAG patients.

Objective of present study was to prospectively establish the prevalence of 25-OH-Vitamin D (25(OH)D) deficiency in a cohort of patients with CAAG. Secondary aims were to evaluate possible association between 25(OH)D levels and histological findings as well as between 25(OH)D and vitamin B12 levels. To the best of our knowledge, this is the first study aimed at investigating the presence of vitamin D deficiency in CAAG patients.

## Methods

From January 2012 to September 2015, 87 patients with histologically confirmed CAAG followed at our Gastroenterology and Endoscopy Unit, Fondazione IRCCS Ca′ Granda Ospedale Maggiore Policlinico of Milan, Italy (16 males and 71 females; mean age 64 ± 13 years) were consecutively evaluated.

Moreover, a control group of 1232 healthy subjects (276 males and 956 females, mean age 62.3 ± 13.2 years) referred to the “Fondazione IRCCS Ca′ Granda Ospedale Maggiore Policlinico of Milan as outpatients for routine laboratory tests was matched for age and gender with the patient group. The criteria for exclusion for both groups were: primary hyperparathyroidism, abnormal calcium values, ongoing vitamin D supplementation or other medication that can interfere with calcium metabolism, renal failure, pancreatic insufficiency, gastrointestinal disease causing malabsorption (e.g. coeliac disease and inflammatory bowel disease), severe hepatic failure, and concomitant malignancy.

All the subjects, after full explanation of the purpose and nature of all procedures used, gave their written informed consent to participate in the study, which was approved by the local Ethics Committee.

All the CAAG patients underwent initial assessment including upper gastrointestinal endoscopy with complete biopsy sample and blood tests with APCA, anti-intrinsic factor antibodies and fasting gastrin determination. The diagnosis of CAAG was established based on the histological confirmation of gastric body mucosal atrophy and/or enterochromaffin-like (ECL) cell hyperplasia, associated with fasting hypergastrinemia and/or presence of APCA or anti-intrinsic factor antibodies.

In case of 25(OH)D deficiency (defined when dealing with values < 20 ng/mL), patients were given cholecalciferol supplementation, with a loading dose of 300,000 U/month for the first 2 months and then 100,000 U every 4 months and interrupted in case of values exceeded 60 ng/ml.

### Laboratory investigations

In all CAAG patients, the levels of total and ionized calcium (Ca^2+^), albumin, phosphate (P), intact PTH, 25(OH)D, creatinine, vitamin B_12_, gastrin and CgA were measured in venous samples obtained after overnight fasting; anticoagulant-free tubes were used for the serum samples and tubes containing EDTA (1 mg/mL of blood) or heparin were utilized for the plasma ones. Serum calcium, albumin, creatinine and urinary calcium and creatinine were measured by standard colorimetric techniques. Total calcium levels were corrected using serum albumin measurements. Plasma ionized calcium was measured using a potentiometric method (Radiometer ABL System 625, Copenhagen, Denmark) on heparinized blood samples within 30 min from blood collection (reference range: 1.15–1.29 mM). Serum intact PTH was measured by chemiluminescence (Elecsys Intact PTH assay, F. Hoffmann-La Roche, Basel, Switzerland) with a sensitivity of 4.0 pg/mL. 25(OH)D was measured by using a commercial available kit (LIAISON® 25-OH Vitamin D TOTAL Assay ref. 310,600, DiaSorin Inc., Stillwater, MN, USA).

### Histological examination

Upper gastrointestinal endoscopy was performed by trained endoscopists using standard endoscopes (Olympus, Japan and Pentax, Japan). Extensive gastric biopsies (2 from the antrum, 2 from the corpus, and 2 from the fundus, larger curvature, in addition to any endoscopically evident mucosal lesion) were obtained in all the cases. Formalin-fixed-paraffin-embedded biopsies were stained with hematoxylin-eosin and Alcian blue–periodic acid-Schiff (PAS) histochemical staining in order to evaluate morphological features. Immunohistochemistry with antibodies anti-chromogranin A, gastrin and Ki-67, when required, was performed using the automatic system DAKO Omnis (Agilent, Santa Clara, California, USA) according to the manufacturer’s instructions. All the samples were evaluated by an experienced pathologist.

The degree of gastritis was classified according to the Sydney classification system [23] which provides guidelines on how to incorporate etiology (i.e. H.pylori, autoimmune), topography (antrum, corpus) and morphological features (chronic inflammation, activity, atrophy and intestinal metaplasia) in pathologist reports of gastric biopsies. More in detail, gastric atrophy defined as loss of appropriate glands, has been classified in mild (reduction of appropriate glands from 1 to 30%), moderate (loss of glands from 31 to 60%) and severe (loss of glands > 60%) (Table 1) [23, 24].

**Table 1:** Atrophy in the gastric mucosa: histological classification and grading [26]

0. Absent (= score 0)				
1. Indefinite (no score is applicable)				
2. Present	Histological type	Location & key lesions		Grading
		Antrum	Corpus	
	2.1. Non-metaplastic	Gland disappearance (shrinking) Fibrosis of the lamina propria		2.1.1. Mild = G1 (1–30%) 2.1.2. Moderate = G2 (31–60%) 2.1.3. Severe = G3 (> 60%)
	2.2. Metaplastic	Metaplasia: – Intestinal	Metaplasia: – Pseudo-pyloric – Intestinal	2.2.1. Mild = G1 (1–30%) 2.2.2. Moderate = G2 (31–60%) 2.2.3. Severe = G3 (> 60%)

The status of the enterochromaffin-like (ECL) cells was classified according to Solcia et al. [25] as: hyperplastic changes in case of ECL cells proliferation < 150 μm (diffuse, linear, micronodular, or adenomatoid hyperplasia); dysplastic lesions for ECL proliferation between 150 and 500 μm (microinvasive lesions, enlarged or fused micronodules, and nodular growth), and neoplasia for ECL proliferation > 500 μm (intramucosal or invasive carcinoids). Minute ECL cell nests characterized by small aggregates less than 50 *μ*m in diameter were separated from true hyperplastic micronodules and were not considered as signs of hyperplasia [26].

### Patients characteristics’

According to the Sydney classification [23], 37 patients had mild, 34 moderate and 16 severe chronic gastritis. The status of the entero-chromaffin-like (ECL) cells was classified according to Solcia et al. [25]. In our series, 24 patients had no cell hyperplasia, 23 had linear hyperplasia and 17 had micronodular hyperplasia, whereas 23 patients had type 1 gastric carcinoids (GC1), of variable size (range 0.2–3 cm), single in 10 cases and multiple in 13.

**Fig. 1:**
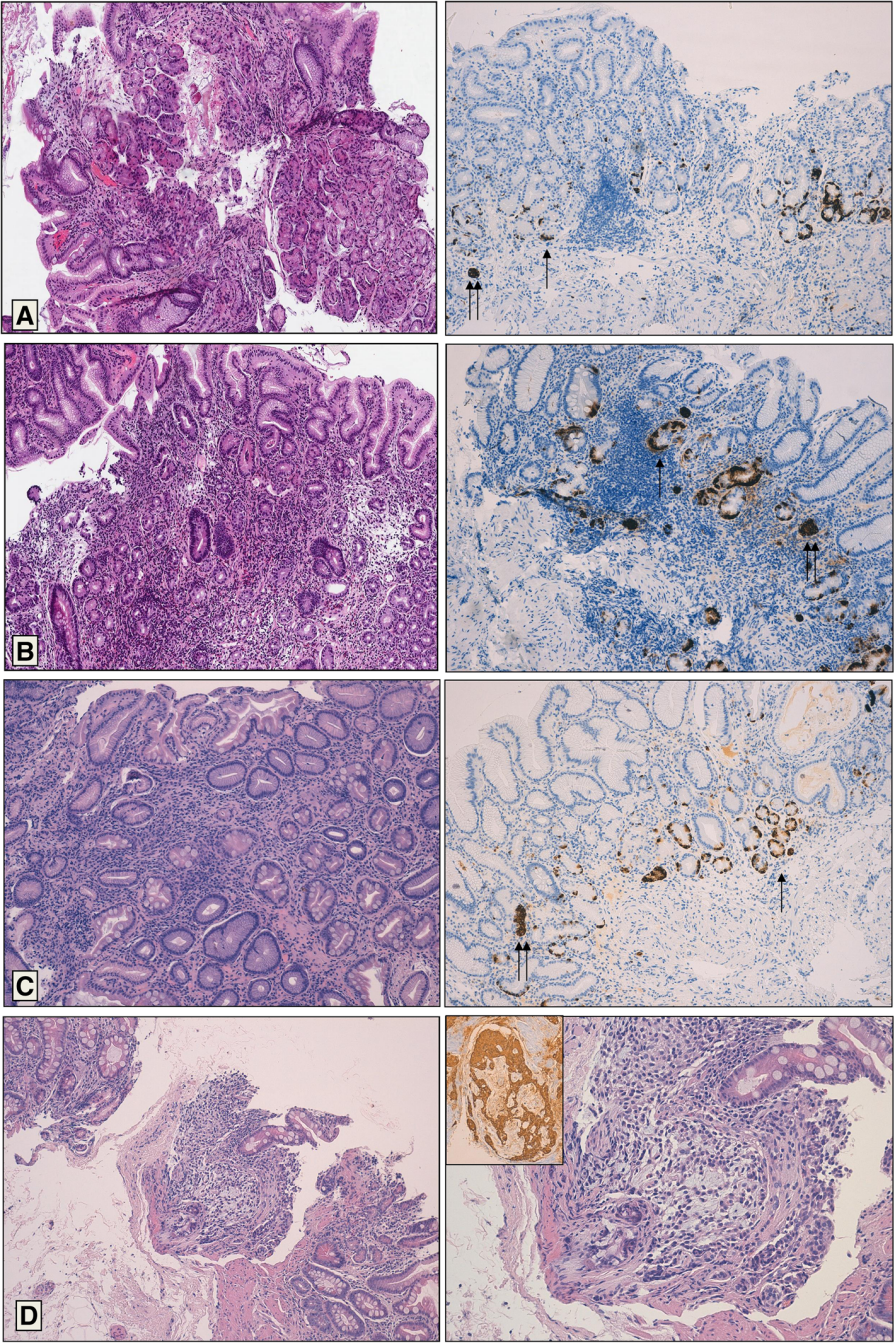
Histological features of atrophic gastritis. **a** Mild atrophic chronic gastritis, showing focal loss of mucosal glands associated with mild chronic inflammation (EE 10x). Chromogranin A immunohistochemical stain shows linear (one arrow) and micronodular (two arrows) neuroendocrine cells hyperplasia. **b** Moderate atrophic chronic gastritis, showing moderate loss of mucosal glands associated with moderate chronic inflammation (EE 10x). Chromogranin A immunohistochemical stain shows linear (one arrow) and micronodular (two arrows) neuroendocrine cells hyperplasia. **c** Severe atrophic chronic gastritis, showing diffuse and severe loss of mucosal glands associated with mild chronic inflammation (EE 10x). Chromogranin A immunohistochemical stain shows linear (one arrow) and micronodular (two arrows) neuroendocrine cells hyperplasia. **d** Gastric carcinoid, characterized by nodular and solid growth pattern of monomorphous neuroendocrine cells (EE 10x left and 20x right), immunoreactive for Chromogranin A (insert)

Representative microphotographs of our series are depicted in Fig. 1.

Anti-parietal cell antibodies (APCA) were present in 79 patients (91%). The associated reported autoimmune diseases were: primary hypothyroidism in 30 patients, vitiligo in four, Graves’ disease in two, mixed connective tissue disease in two, multiple sclerosis, psoriasis, alopecia, PGA type 1, myasthenia gravis and scleroderma were reported in one patient each. Previous *Helicobacter pylori* (*H. pylori*) infection was reported in 20 CAAG patients, all of them having been treated with successful eradication. Active infection was present in two patients, who have been treated successfully.

Among the CAAG patients, dual-energy X-ray absorptiometry (DXA) to evaluate bone mineral density (BMD) (g/cm^2^) was available for 39 out of 87 patients. Lumbar spine BMD was measured using the average value for L1 to L4. Femur BMD was measured at femoral neck. Low bone density and osteoporosis were diagnosed using the World Health Organization criteria (− 2.5 < T-score < − 1.0 and T-score ≤ − 2.5 respectively).

### Statistical analysis

Continuous variables were reported as mean ± standard deviation (SD) or median and range; categorical variables were reported as count (percentage). All data were tested for distribution normality via the Kolmogoroff-Smirnoff test. The differences between groups were assessed with the Mann-Whitney and Kruskal-Wallis test as appropriate. Differences between percentages were evaluated by Fisher’s exact test. The relationships between variables were determined by Spearman’s coefficient. A *p* value < 0.05, two-sided, was considered statistically significant. The analyses were carried out by software Graph Pad Prism version 5.00 and Graph Pad State Mat version 2, for Windows (GraphPad Software, San Diego, California, USA).

## Results

The CAAG group showed significantly lower 25(OH)D mean levels (18.8 ± 9.7 ng/ml) compared with the control group (27.0 ± 16.3 ng/ml) (*p* < 0.0001). 25(OH)D deficiency, defined as 25(OH)D levels lower than 20 ng/ml, was observed in 57 (66%) of the 87 CAAG patients and in 438 (36%) of the patients in the control group, (Fisher’s exact test *p* < 0.0001) (Table 2).

**Table 2:** Demographic and biochemical data of CAAG patients and healthy controls

	CAAG patients (*n* = 87)	Healthy control (*n* = 1232)	*p* value
Female (%)	71 (82)	956 (76)	ns
Age (years) Mean ± SD	63.5 ± 12.8	62.3 ± 13.2	ns
25(OH)D ng/ml Mean ± SD	18.8 ± 9.7	27.0 ± 16.3	< 0.0001
25(OH)D < 20 ng/ml n (%)	57 (66%)	438 (36%)	< 0.0001
25(OH)D < 12.5 ng/ml n (%)	27 (31%)	160 (13%)	< 0.0001

*CAAG* chronic autoimmune atrophic gastritis, *25(OH)D* 25-OH-Vitamin D, *ns* not significant

25(OH)D levels lower than 12.5 ng/ml, was present in 27 of 87 patients (31%) in the CAAG group and in 160 of 1232 (13%) patients in the control group (Fisher’s exact test *p* < 0.0001). The mean 25(OH)D values were not significantly different between female and male patients, neither among CAAG patients (female mean 19.2 vs. male 17.0 ng/ml, *p* = 0.65) nor among controls (female mean 26.9 vs. male 27.0 ng/ml, *p* = 0.07). A statistical difference in the number of patients diagnosed in summer/spring vs winter/autumn was not observed (*p* < 0.001).

The demographic and biochemical data of CAAG patients with and without vitamin D deficiency are detailed in Table 3.

**Table 3:** Demographic and biochemical data of CAAG patients with and without vitamin D deficiency

Patients	25(OH)D deficiency (*n* = 57)	Normal 25(OH)D (*n* = 30)	*p* value
Female n (%)	47 (83)	24 (80)	ns
Age (years) Mean ± SD	63.4 ± 12.8	64.6 ± 12.9	ns
APCA positivity n (%)	50 (88)	29 (97)	ns
Atrophy n (%)			
• mild	24 (42)	13 (43)	ns
• moderate	20 (35)	14 (47)	
• severe	13 (23)	3 (10)	
ECL cell hyperplasia n (%)			
• normal	17 (30)	7 (23)	ns
• linear	12 (21)	11 (37)	
• micronodular	10 (17)	7 (23)	
• gastric carcinoid	18 (32)	5 (17)	

*APCA* Anti-parietal cell antibodies, *ECL* entero-chromaffin-like, *25(OH)D* 25-OH-Vitamin D

In CAAG patients a deficit of vitamin B12, defined as vitamin B12 levels below 190 ng/ml, was present in 28 cases (32%). In this setting, a significant correlation between vitamin B_12_ values at diagnosis and 25(OH)D levels was observed (r_s_ = 0.25, *p* = 0.01). In detail, mean 25(OH)D was lower in patients with vitamin B_12_ deficiency (15.9 ± 9.9 ng/ml) compared with patients without vitamin B_12_ deficiency (20.2 ± 9.7 ng/ml) (*p* = 0.025).

All the CAAG patients had normal serum calcium levels (mean 9.6 ± 1.8 mg/dl) and ionized calcium levels (1.2 ± 0.1 mmol/l). Elevation in PTH levels, defined as PTH levels > 65 pg/ml was observed in 22 of the 87 CAAG patients (25%) (mean 74 ± 24.2 pg/ml). In all these cases, proper vitamin D supplementation led to PTH levels normalization, thus the diagnosis of secondary hyperparathyroidism due to vitamin D deficiency was made.

**Fig. 2:**
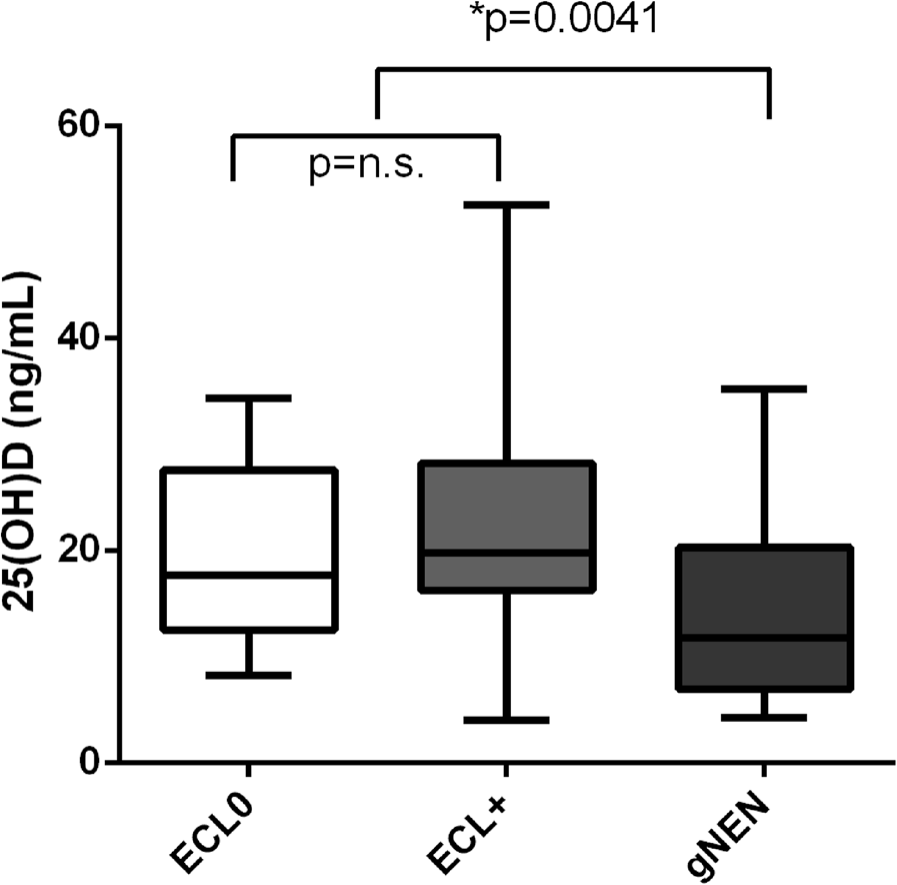
25(OH)D levels in CAAG patients having absence of ECL hyperplasia (ECL0), linear or micronodular ECL hyperplasia (ECL+) or gastric carcinoid (GC1)

In our series, the CAAG patients with moderate to severe gastric atrophy at histology presented significantly lower 25(OH)D values than those with mild atrophy (11.8 vs. 20 ng/ml; *p* = 0.0047). Moreover, 25(OH)D levels were significantly lower in CAAG patients with gastric carcinoid as compared to the group of CAAG patients without ECL hyperplasia or having linear or micronodular ECL hyperplasia (11.80 vs. 19.75 ng/ml; *p* = 0.0041) (Fig. 2).

A significant inverse correlation (r_s_ = − 0.25, *p* = 0.032) between 25(OH)D and BMI was observed in CAAG patients. A direct correlation was observed between 25(OH)D and circulating vitamin B_12_ (r_s_ = − 0.28, *p* = 0.009), whereas there was not any significant correlation between 25(OH)D levels and gastrin levels (*p* = 0.7). Among the 39 CAAG patients who underwent DXA, 17 patients had osteoporosis, 14 had low bone density/osteopenia, while in the remaining 5 patients the DXA scan was normal. 25(OH)D levels did not significantly differ among patients with osteoporosis/osteopenia and normal DXA.

## Discussion

The present study has documented significantly lower mean 25(OH)D values in CAAG patients as compared with outpatient controls. Moreover, in CAAG patients a significantly higher rate of 25(OH)D deficiency, considering 25(OH)D values lower either than 20 ng/ml or 12.5 ng/ml, was observed. These results are similar to those observed by Antico et al. [22] that reported lower 25(OH)D values in CAAG patients when compared to patients with non-specific gastritis or healthy controls. The authors reported 25(OH)D mean levels in CAAG patients to be 9.8 ± 5.6 ng/mL versus 21.3 ± 12.2 ng/mL in controls. On the basis of their results, the authors speculated that vitamin D deficiency may play a role in the pathogenesis of the autoimmune processes [22]. In addition a previous study from our group [21] observed an higher rate of hyperparathyroidism secondary to vitamin D deficiency in CAAG patients. On the other hand, Eastell et al. [20] did not show a significant difference in 25(OH)D values among 21 CAAG patients and healthy controls [20], even though the low number of cases evaluated may have limited the results of their study.

To date the pathogenesis of hypovitaminosis D in CAAG patients has not been clarified. However, as for other micronutrients [11], a decreased absorption/increased destruction of vitamin D in the gastrointestinal mucosa due to the hypochlorhydria and bacterial overgrowth might be hypothesized. Interestingly, this is the first study showing significantly lower 25(OH)D values in CAAG with moderate to severe gastric atrophy as compared to those with mild atrophy (*p* = 0.0047). This finding strongly suggests a causal association between the degree of mucosal atrophy and 25(OH)D levels. It may be postulated that in patients with mild atrophy the residual production of gastric acid can preserve a sufficient rate of vitamin D absorption, which, however, becomes insufficient in more advanced stages of the disease.

The hypothesis of a progressive functional alteration in the gastric mucosa over time has been already proposed with regard to patients with CAAG so as to explain the progression from microcytic anemia to macrocytic anemia [9, 27]. Indeed, CAAG patients with iron deficiency anemia have been reported to be younger than patients presenting with pernicious anemia. This suggests iron deficiency to be an early manifestation of CAAG, while the depletion of vitamin B_12_ stores appears to take many years longer and to reveal in older patients, when a severe deficiency of the intrinsic factor has been established [9, 27]. Accordingly, in our study CAAG patients’ presented a significant correlation between vitamin B_12_ values at diagnosis and 25(OH)D levels (*p* = 0.009), probably because these levels are both dependent on the residual gastric function and are reduced in the advanced stages of disease.

Interestingly, our study has showed significantly lower 25-OHvitD levels in patients with gastric neuroendocrine neoplasms (NENs) as compared to patients without gastric NENs. The pathogenic mechanism leading to this association has not been fully elucidated yet, however vitamin D has proved to be involved in cell growth, apoptosis, differentiation, cell adhesion, immune regulation, angiogenesis, and metastasis in epithelial tissues [28, 29] Therefore, the possible role of hypovitaminosis D in the development of gastric NENs may be postulated. In the last few years a number of studies have focused on the antineoplastic properties of vitamin D in different solid neoplasms [30–32] and recently a paper from our group showed a significant higher prevalence of vitamin D deficiency among NEN patients [33]. Moreover, in the same study we observed an improved clinical outcome for patients supplemented by vitamin D, reinforcing the hypothesis on an antiproliferative effect of vitamin D supplementation on NEN.

Gastric achlorhydria secondary to gastric surgery, long-term PPI intake or CAAG has been reported to increase the risk of bone health impairment [14]. A recent meta-analysis has shown that PPIs increase the rate of any site fractures of 16% and in particular the risk of hip (30%) and spine fractures (56%) [34]. However, to date the studies investigating the occurrence of osteoporosis in the setting of CAAG are rare and inconclusive [20]. In the present series, DXA scan results did not significantly differ between CAAG patients with vitamin D deficiency and those with normal levels. However, this can be owed to the small number of patients who underwent bone densitometry in the present study (39 out of 86). Therefore, the exact influence of vitamin D deficiency on osteopenia/osteoporosis in CAAG patients remains to be accurately evaluated. Some previous reports have suggested a reduction of bone density in the lumbar spine of patients with pernicious anemia, even though negative reports have also been published [35–37]. A recent study by Kim et al. has found a significant association between atrophic gastritis and osteoporosis in post-menopausal women aged 60 or older, after adjusting for age, body mass index, triglycerides, cholesterol, alcohol consumption, and smoking status [16]. Interestingly, a previous study had not found a significant association between bone mass density and atrophic gastritis [38], however, the participants in that study were relatively young. Therefore, it is possible that the onset of 25(OH)D deficiency in patients with CAAG is a long-developing process leading to significant alterations in older patients with a long-standing history of disease.

A decrease in calcium salts dissolution and absorption in non-acidic conditions has been previously suggested as the main pathological mechanism for bone impairment in patients with achlorhydria. Remarkably, the active transcellular absorption of ionized calcium in the duodenum and proximal small intestine represents the most important physiological pathway for calcium absorption and is highly dependent on vitamin D. Thus, it seems possible that the vitamin D deficiency in CAAG patients also explains calcium malabsorption and alterations in bone mineralization.

Possible limitations of our study are the relatively small number of patients evaluated and that 25(OH)D determination were obtained during outpatient’s examination during all the year; however we did not observe a statistical difference in the number of patients diagnosed in summer/spring vs winter/autumn. Strengths of presents study were the use of strict diagnostic criteria for CAAG which enables to obtain a highly homogenous group of study and the centralization of all laboratory tests.

## Conclusions

This study has clearly demonstrated that vitamin D deficiency is more frequent in patients affected by CAAG than in the general population and correlates with the grade of gastric atrophy. The pathogenic mechanism underlying this association has not been fully elucidated, but it is probably due to a decreased absorption of vitamin D secondary to gastric hypo-achlorhydria. Further larger studies are necessary to evaluate the relevance of vitamin D deficiency in the occurrence of osteopenia and osteoporosis in CAAG patients.

## Abbreviations

25(OH)D: 25-OH-Vitamin D
APCA: Anti-parietal cell antibodies
BMD: Bone mineral density
BMI: Body mass index
Ca^2+^: Ionized calcium
CAAG: Chronic autoimmune atrophic gastritis
CgA: Chromogranin A
DXA: Dual-energy X-ray absorptiometry
ECL: Entero-chromaffin-like
GC1: Type 1 gastric carcinoids
NENs: Neuroendocrine neoplasms
P: Phosphate
PTH: Parathormone
